# Allometric relationships between leaf and petiole traits across 31 floating-leaved plants reveal a different adaptation pattern from terrestrial plants

**DOI:** 10.1093/aob/mcad007

**Published:** 2023-01-19

**Authors:** Yang Liu, Hui Liu, Lars Baastrup-Spohr, Zhizhong Li, Wei Li, Junfeng Pan, Yu Cao

**Affiliations:** Aquatic Plants Research Center, Wuhan Botanical Garden, Chinese Academy of Sciences, Wuhan 430074, China; University of Chinese Academy of Sciences, Beijing 100049, China; Freshwater Biological Laboratory, Department of Biology, University of Copenhagen, Copenhagen 2100, Denmark; Key Laboratory of Vegetation Restoration and Management of Degraded Ecosystem, South China Botanical Garden, Chinese Academy of Sciences, Guangzhou 510650, China; South China National Botanical Garden, Guangzhou 510650, China; Freshwater Biological Laboratory, Department of Biology, University of Copenhagen, Copenhagen 2100, Denmark; Aquatic Plants Research Center, Wuhan Botanical Garden, Chinese Academy of Sciences, Wuhan 430074, China; Research Center for Ecology, College of Science, Tibet University, Lhasa 850000, China; Aquatic Plants Research Center, Wuhan Botanical Garden, Chinese Academy of Sciences, Wuhan 430074, China; Center for Plant Ecology, Core Botanical Gardens, Chinese Academy of Sciences, Wuhan 430074, China; Horticulture and Conservation Centre, Wuhan Botanical Garden, Chinese Academy of Sciences, Wuhan 430074, China; Aquatic Plants Research Center, Wuhan Botanical Garden, Chinese Academy of Sciences, Wuhan 430074, China

**Keywords:** Stoma, xylem, vascular, water transport, phylogeny, evolution, aquatic plant

## Abstract

**Background and Aims:**

Allometric scaling between stomata and xylem for terrestrial woody plants is a widely observed pattern that may be constrained by water transport. Floating-leaved plants, a particular life form of aquatic plants, have leaves in direct contact with both air and water and a poorly developed xylem that may not be limited by water supply as for terrestrial plants. However, whether such an allometric scaling relationship still exists in floating-leaved plants has not been explored.

**Methods:**

We analysed 31 floating-leaved species/varieties with a range in leaf area covering six orders of magnitude. For all 31 floating-leaved plants, we studied the allometric relationships between leaf area and petiole transverse area, and between total stomatal area and petiole vascular area.

**Key Results:**

The slopes of both relationships were similar to the slope of the allometric relationship (1.23) between total stomatal area and xylem area of 53 terrestrial plants. However, for ten of them with xylem that can be clearly defined, the strong positive relationship between total stomatal area and petiole xylem area had a significantly smaller slope than that of terrestrial plants (0.64 vs. 1.23). Furthermore, after considering phylogeny, the scaling relationships between total stomatal area and petiole traits in floating-leaved plants remained significant.

**Conclusions:**

We speculated that for floating-leaved plants, the hyperallometric relationship (slope >1) between the construction of leaf/stoma and petiole was promoted by the high demand for photosynthesis and thus more leaves/stomata. While the hypoallometric relationship (slope <1) between stomatal and xylem area was related more to hydraulic processes, the selection pressure on stomata was lower than xylem of floating-leaved plants. Allometric relationships among the hydraulic traits on water transport of aquatic plants are the result of natural selection to achieve maximum carbon gain, which is similar to terrestrial plants.

## INTRODUCTION

Water transport is one of the most important processes for plant growth. In terrestrial plants, water deficiency is considered as a critical factor limiting plant growth and affecting plant survival. Higher plants often rely on specialized tissues to deal with water transport; i.e., the shift of tracheid to xylem vessels greatly improves the efficiency of long-distance water transport, and stomata control the rate of gas exchange that strongly affects water loss (Hetherington and Woodward, 2003; Hölttä and Sperry, 2014). Moreover, allometric scaling was found to regulate water deficiency at both a plant level and a leaf level (Brodribb *et al.*, 2005; Zhong *et al.*, 2019; Xiong and Nadal, 2020). Allometric relationships between leaf stomata and related tissues (e.g. xylem) are expected to achieve the maximum gain at the minimum energy cost (e.g. construction of stem vs. leaf) during the process of water transport, which has shaped the evolution of plant function across vascular plants (Enquist, 2002, 2003; Coomes *et al.*, 2008; Carins *et al.*, 2014; Wen *et al.*, 2020). For example, Zhong *et al.* (2020) found that the balance of water exchange between liquid and gas phases depended on the consistent scaling between leaf total stomatal area and stem xylem area in seedlings of woody terrestrial species. Allometric relationships between photosynthetic, autotrophic tissues (e.g. leaves, stomata) and vascular tissue (e.g. xylem) in plants are the result of natural selection on the optimization of carbon costs and nutrient allocation in plants (West *et al.*, 1999; Enquist, 2002). In addition, the hyperallometric scaling (slope significantly greater than 1; see Shingleton *et al.*, 2009) between stomatal and xylem traits showed that stomata scale to xylem exponentially, and a proportionally larger increase in leaf stomata area is expected for the increase in xylem area per unit (Zhong *et al.*, 2019, 2020).

**Fig. 1.**
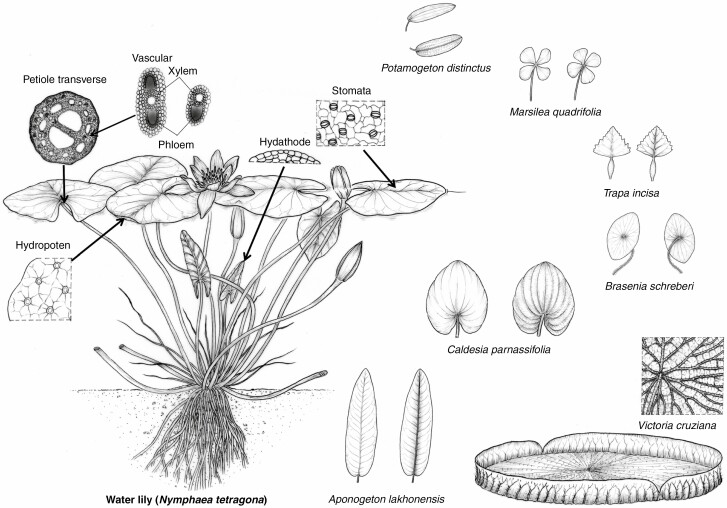
Diagrams illustrating organs and tissues that are related to water transport in a water lily and other experimental species.

Aquatic plants usually have an adequate water supply, a degraded vascular system, and an investment reduction compared to terrestrial plants (Sculthorpe, 1967). As a special life form in aquatic plants, floating-leaved species have leaves with the upper (adaxial) surface in air and the lower (abaxial) surface in water, and in contrast to terrestrial plants, stomata of floating-leaved plants are mainly distributed in the upper (adaxial) surface of leaves (Sculthorpe, 1967). Previous studies have found that they possess similar stomatal density (e.g. *Potamogeton fryeri*), but tracheid or poorly developed xylem and intensified aerenchyma in petiole compared to terrestrial plants (Sculthorpe, 1967; Doležal *et al.*, 2021; Han *et al.*, 2021). More intriguingly, floating-leaved plants possess hydathodes – pores used for water excretion on the leaf margin – and hydropotens – specialized structures on the leaf lower (abaxial) surface that are assumed to directly absorb water from the aquatic environment (Fig. 1). Both structures participate in water exchange processes between the leaf and atmosphere (Mortlock, 1952; Stevens, 1956; Sculthorpe, 1967). However, hydathodes are active only in young leaves, and they become blocked and functionless during leaf development and usually do not play a role in the water transport of mature leaves (Mortlock, 1952; Stevens, 1956). Meanwhile, floating-leaved plants lie on the water surface and cannot flexibly adjust leaf angles to change water or light conditions, and thus they can only regulate water transport and gas exchange processes by adjusting structural traits such as stomata numbers. Additionally, many floating-leaved plants do not have the standing stems of terrestrial plants, such that scaling relationships between leaf and petiole traits are more important in investigating their growth and allocation. Therefore, given that the environmental constraints on the allometric relationships between stomata and petiole vascular traits of terrestrial plants are relieved for floating-leaved plants, we may expect different patterns for floating-leaved plants compared to those found in terrestrial species.

However, there may still be another possibility that aquatic plants keep similar allometric relationships to their terrestrial relatives, because aquatic angiosperms have undergone hundreds of events of plant ‘regression’ evolution from terrestrial ancestors (Sculthorpe, 1967; Cook, 1988; Les, 2015; Meseguer *et al.*, 2022), probably leading to a high phylogenetic conservatism in stomata and petiole traits of aquatic plants and similar allometric relationships to their terrestrial relatives (Liu *et al.*, 2015; Chen *et al.*, 2022). Thus, comparative analyses on the allometric scaling across species should consider phylogeny, and we do not know whether the allometric scaling between leaf stomata and petiole xylem traits of floating-leaved plants still exist despite the different phylogenetic backgrounds.

Here, we analysed 31 floating-leaved species/varieties to study the relationship between leaf and petiole traits of floating leaves. We hypothesized that slopes or intercepts of allometric relationships between stomatal and petiole traits across floating-leaved plants might differ from terrestrial plants, given their distinctive structural (hydropotens, stomata location) and environmental differences (water availability) from terrestrial plants.

## MATERIALS AND METHODS

Thirty-one floating-leaved species (including three varieties, herein referred to as species for convenience) were collected from outdoor shallow ponds or tanks in Wuhan Botanical Garden from 23 June to 24 August, 2020 and from 9 to 14 July, 2021. The ponds or tanks are ca. 50 cm deep with light reaching the bottom and filled with water and sediment from the nearby Donghu Lake. Details of the studied species are provided in Supplementary Data Table S1. Species names were verified according to www.theplantlist.org, https://powo.science.kew.org/ and www.iwgs.org. In 2020, at least ten healthy similar-sized mature floating leaves with petioles of similar size were collected for 26 species to determine leaf stomatal and petiole vascular traits following Zhong *et al.* (2020). In 2021, an additional five floating-leaved plants were collected and measured.

### Leaf stomatal traits

Leaf area was calculated from digital photographs using AreaAna software (Huazhong University of Sciences and Technology, China). A square segment of the leaf with an area of 50–100 mm^2^ in the central of the leaf was cut to determine the stomatal traits. Leaf stomatal traits, including stomatal size (μm^2^) and stomatal number, were measured on the upper (adaxial) surface (Cornelissen *et al.*, 2003; Zhong *et al.*, 2020). The stomatal number of each of six randomly selected views (0.148 mm^2^ at 40× magnification) was counted on each segment and averaged for each leaf. Stomatal size was defined as the area enclosed by two guard cells; i.e. stomatal size = (*a*/2)*b*π, where *a* and *b* are the maximum length and maximum width of the guard cell of six randomly selected stomata (Cornelissen *et al.*, 2003). Stomatal density (mm^−2^) was calculated as the stomatal number on each view divided by 0.148 mm^2^ (leaf area of one scope view). Total stomatal number was defined as stomatal density multiplied by leaf area. Similarly, total stomatal area (mm^2^) was calculated by multiplying stomatal density by leaf area and stomatal size. The stomatal area unit (total stomatal area per unit of area) refers to total stomatal area divided by leaf area.

### Petiole traits

Transverse sections were taken at the petiole close to the base of the blade and inspected with a light microscope (Motic BA310) using Motic Image Plus 2.0 ML software. The petiole vascular and xylem areas were manually encircled and calculated using the same software. The division of xylem and phloem is not feasible for many of the studied species because the morphological features of xylem and phloem are mixed (Sculthorpe, 1967), and thus petiole xylem area was determined only for ten species of in which xylem can be clearly defined (detailed in Supplementary Data Table S1), and five replicates for each species. We found a strong correlation between petiole xylem area and vascular area for the ten species (*r*^2^ = 0.89, *P* < 0.001; Fig. S1), such that we used petiole vascular area to represent petiole xylem area in the following analyses.

Terrestrial plant traits, including total stomata area and stem xylem area, were collected from previous studies (Zhong *et al.*, 2019, 2020). The ratio of stomatal area to xylem area (*A*_s_/*A*_x_) was defined as the total stomatal area divided by the transverse area of the xylem.

### Phylogenetic signal detection

We applied phylogenetic analyses to determine whether allometric relationships between stomatal and petiole traits across 31 floating-leaved species were affected by phylogeny. The phylogenetic relationships of the 31 species were obtained using phylomatic-awk-1.1.0 (zanne2014.new) based on Zanne *et al.* (2014). We extracted the phylogenetic information of the chosen species from the backbone tree in Zanne *et al.* (2014), which was built based on multi-gene molecular and fossil data.

### Statistical analyses

The phylogenetic signals of plant traits were tested in the function multiPhylosignal in the package ‘picante’, and Blomberg’s *K* was used to compare the observed value of the trait with the predicted value of the Brownian motion model (Blomberg *et al.*, 2003). A value of *K* = 0 indicates no phylogenetic signal, and *K* > 1 suggests stronger similarities among closely related species than expected under Brownian motion (Blomberg *et al.*, 2003).

Relationships between different stomatal traits (e.g. stomatal size and stomatal density), and relationships between stomatal traits and petiole traits were analysed using generalized least squares (gls) methods. We constructed models considering or not considering the phylogeny or not; i.e. gls (Stomatal trait ~ Petiole traits, data = all measurement table) when not including phylogenetic relationships, and pgls [Stomatal traits ~ Petiole traits, correlation = corBrownian(value = 1, phy = tree built above), data = all measurement table] when including phylogenetic relationships.

Analysis of covariance (ANCOVA) was performed to test whether the slopes of the correlation models between leaf and petiole traits differed between floating-leaved plants and terrestrial plants. Standardized major axis (SMA) regressions were conducted with the ‘SMATR’ package in R to test whether slopes were different from 1 or slopes differ between groups (floating-leaved plants and terrestrial plants in our case) (Warton *et al.*, 2006).

When studying the allometric relationships, data were generally log-transformed to better represent the biological variation as a linear relationship, because the raw data always show an exponential curve because many biological phenomena (e.g. growth, reproduction) are multiplicative fundamentally (Kerkhoff and Enquist, 2009). At the same time, log-transformed data can satisfy the assumption of normal distribution required by the pgls model we used.

To compare *A*_s_/*A*_x_ between terrestrial and floating-leaved plants, we performed a non-parametric test [Mann–Whitney U test, Wilcox test (paired = False)] since the variance is not homogeneous.

All statistical analyses were performed using R software (version 4.1.2).

## RESULTS

Across 31 floating-leaved species, values of leaf and petiole traits covered six orders of magnitude. Leaf area ranged from 0.74 ± 0.39 cm^2^ (*Cabomba caroliniana*) to 6530.63 ± 3173.09 cm^2^ (*Victoria cruziana*), and petiole transverse area varied from 0.36 ± 0.19 mm^2^ (*Potamogeton natans*) to 123.11 ± 22.02 mm^2^ (*V. cruziana*).

We also found substantial variation across the 31 species in stomatal traits: stomatal size varied from 84.32 ± 19.12 µm^2^ in *Nelumbo nucifera* to 2574.61 ± 164.36 µm^2^ in *Sagittaria natans*; stomatal density ranged from 36.49 ± 3.32 to 534.91 ± 18.89 mm^−2^; and total stomatal area varied from 1.43 ± 0.72 to 80 066.97 ± 53 032.96 mm^2^. Further analysis showed that stomatal size was negatively correlated with stomatal density (*r*^2^ = 0.66, *P* < 0.001; Supplementary Data Fig. S2a).

The smallest petiole vascular area was 0.02 ± 0.01 mm^2^ in *C. caroliniana*, while the largest was 9.31 ± 2.95 mm^2^ in *V. cruziana* across the 31 species. For the chosen ten species included in the determination of petiole xylem area, xylem area in the petiole ranged from 0.03 ± 0.01 mm^2^ in *Hydrocharis dubia* to 1.78 ± 1.00 mm^2^ in *N. nucifera*. In addition, the ratio of stomatal area to xylem area (*A*_s_/*A*_x_) of the ten floating-leaved plants was 4493 ± 3034, with mean values ranging from 1761 to 8237 for each species, ca. 30 times larger than the mean values (145.03 ± 167.20) in terrestrial plants (*W* = 2628, *P* < 0.001).

**Fig. 2.**
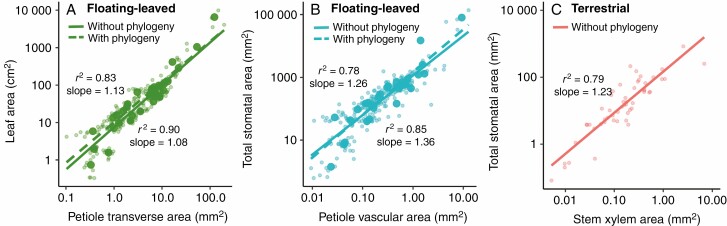
The scaling relationships between leaf and petiole traits: (A) between leaf area and petiole transverse area for floating-leaved plants (green); (B) between leaf stomata area and petiole vascular area (blue) for floating-leaved plants; (C) between leaf stomatal area and stem xylem area for terrestrial plants (orange). Both *x*-axis and *y*-axis are log–log scaled. Large dots represent the mean value for each species in floating-leaved plants and the small transparent points represent the mean value for each leaf sample. The solid line (*r*^2^ and slope values on the left) indicates the condition without considering phylogenetic signal, and the dashed line (*r*^2^ and slope values on the right) that when considering phylogenetic signal.

**Fig. 3.**
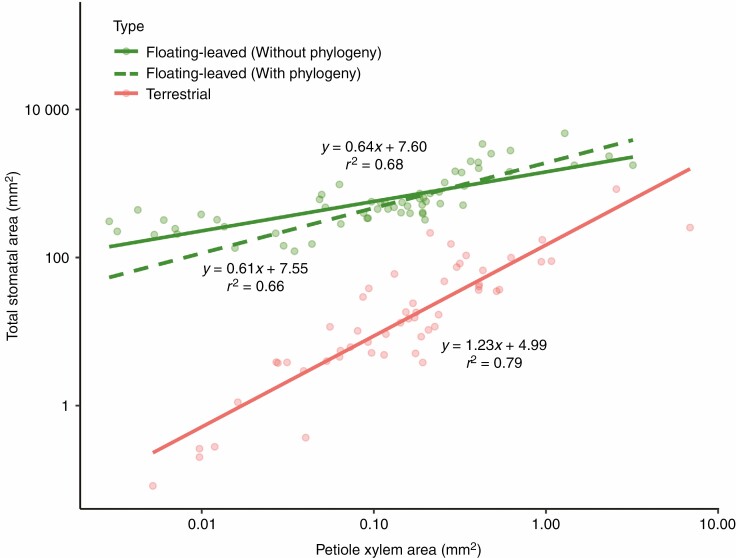
The scaling relationships between total stomatal area and petiole xylem area across ten floating-leaved plants and 53 terrestrial woody plants (Zhong *et al.*, 2020). Both *x*-axis and *y*-axis are log–log scaled. ‘Floating-leaved’ (green) represents floating-leaved plants, and ‘terrestrial’ (orange) represents terrestrial plants. Large green dots represent the mean value for each species in floating-leaved plants. The solid line (formula and *r*^2^ above lines) for floating-leaved plants indicates the condition without considering phylogenetic analysis, and the dashed line (formula and *r*^2^ below lines) that when considering phylogenetic analysis.

A scaling relationship across the 31 floating-leaved species was found between leaf area and petiole transverse area on a log-based regression (*r*^2^ = 0.83, *P* < 0.001, Fig. 2A), suggesting that leaf area is allometric to petiole transverse area. Moreover, the correlation between stomatal and petiole traits showed that most stomatal traits, especially total stomatal area (*r*^2^ = 0.78, *P* < 0.001, Fig. 2B), were positively and significantly related to petiole vascular area. Both slopes (1.13 ± 0.03 in Fig. 2A and 1.26 ± 0.04 in Fig. 2B) of regression models for the 31 floating-leaved species were notably larger than the slope of 1 (allometry) (SMA; Supplementary Data Table S2). Furthermore, these slopes did not differ significantly from that (1.23 ± 0.09) of regression between total stomatal and stem xylem area across 53 terrestrial plants (ANCOVA, both *F* < 1.67, *P* > 0.05; Fig. 2; Table S2). Additionally, total stomatal area was significantly correlated with petiole xylem area across ten floating-leaved plants (*r*^2^ = 0.68, *P* < 0.001, Fig. 3), with a significantly smaller slope but a significantly higher intercept than that of terrestrial plants (ANCOVA, both *F* > 41.13, *P* < 0.001; Fig. 3; Table S2). Also, a positive relationship was detected between petiole xylem area and stomatal density (*r*^2^ = 0.24, *P* < 0.001; Fig. S2B) in ten floating-leaved plants.

Phylogenetic signal analysis of these traits revealed weak signals in stomatal size (*K* = 0.41, *P* = 0.028) and stomatal area per unit (*K* = 0.42, *P* = 0.057), but no phylogenetic signals in other stomatal traits and petiole traits (Supplementary Data Table S3). The insignificant relationship between stomatal size and petiole vascular area was marginally significant after including a phylogenetic relationship (*F* = 4.40, *P* = 0.046; Table 1). Moreover, scaling relationships between most leaf (stomata) traits and petiole traits were unchanged after considering phylogeny (Table 1).

**Table 1.** The results of correlation analysis between stomatal traits and petiole traits by considering or not considering the phylogenetic relationship with the mean value of each species/variety

Petiole traits	Model	Stomatal size			Stomatal density			Stomatal area unit			Total stomatal area			Total stomatal number		
		Slope	*F*	*P*	Slope	*F*	*P*	Slope	*F*	*P*	Slope	*F*	*P*	Slope	*F*	*P*
Vascular area (*n* = 31)	Without phylogeny	−0.11	1.19	NS	0.36	16.69	***	0.14	5.38	*	1.44	153.51	***	1.54	128.55	***
	With phylogeny	−0.30	4.40	*	0.38	15.95	***	0.02	0.04	NS	1.36	84.26	***	1.64	117.69	***
Xylem area (*n* = 10)	Without phylogeny	−0.56	6.86	*	0.37	3.25	NS	−0.19	2.79	NS	0.59	15.58	**	1.14	17.76	**
	With phylogeny	−0.30	0.66	NS	0.27	0.30	NS	−0.03	0.02	NS	0.61	10.00	*	0.91	7.10	*

All traits are log-transformed. Significance levels: NS, *P* > 0.05; *P < 0.05; **P < 0.01; ***P < 0.001.

## DISCUSSION

This study is the first attempt to show evidence of the scaling relationship between leaf and petiole traits in aquatic plants. Consistent with our hypothesis, significant allometric relationships were uncovered between leaf stomata and petiole xylem (and vascular) area across 31 floating-leaved plants, and the slope of the correlation between total stomatal area and petiole xylem area was significantly smaller than that of terrestrial plants. However, the scaling relationships between total stomatal area and petiole xylem (or vascular) area in floating-leaved plants remained significantly correlated when considering phylogeny. The new allometric scaling relationship between total stomatal area and petiole xylem area, different from terrestrial plants, indicated different water–carbon coupling strategies (Gleason *et al.*, 2018; Zhong *et al.*, 2019) and probably an independent evolutionary allometry in floating-leaved plants.

The slope of the relationship between total stomatal area and petiole xylem area was remarkably smaller than 1 (slope = 0.64, hypoallometry). This implied a lessening selection pressure on stomata but a stronger selection pressure on the xylem of aquatic plants than terrestrial plants. Moreover, the small slope for floating-leaved plants also revealed that the role of total stomatal area and petiole xylem area for floating-leaved plants no longer requires strong water loss control. Furthermore, compared with terrestrial plants, the slope between stomatal and xylem traits was significantly smaller (0.64 vs. 1.23) and there was a larger ratio of stomatal area and xylem area (*A*_s_/*A*_x_) for floating leaves (4493.30 vs. 145.03), implying that there may be other water supply sources or higher hydraulic conductance for floating-leaved plants to balance water loss from stomata. Also of note was that higher total stomatal area for a given petiole xylem area might be influenced by the higher thickness of floating-leaved leaves as a larger stomata area is needed to provide mechanical support for the larger leaf volume in comparison with the thin leaves of terrestrial plants. Moreover, a larger intercept of the regression between total stomatal area and petiole xylem area of floating-leaved plants than that of terrestrial plants suggests that floating-leaved plants need larger stomatal areas at a given petiole xylem area across a wide range of leaf size. This indicated that sufficient water supply ensures that floating-leaved plants have the opportunity to capture more carbon. However, the xylem in the petiole of floating-leaved plants has less capacity to transport water than that in woody plants as the vascular tissue in floating-leaved plants includes simplified xylem and phloem (Sculthorpe, 1967). For those in Alismaceae, Butomaceae, Potamogetonaceae and Nymphaeaceae, the xylem comprises only annular and spiral-thickened tracheids (Sculthorpe, 1967), while for *Trapa acornis*, vessels are degenerated gradually from the stem top to base (Yan *et al.*, 1993). Therefore, water supply directly through the xylem was strongly weakened in floating-leaved species. Next, according to Zhong *et al.* (2020), the allometric scaling between stoma and xylem in seedlings of woody species is the most cost-effective for the balance of water exchange between leaf stomata (gas phase) and other related tissues (e.g. xylem, liquid phase). If floating-leaved plants follow a similar rule, their degraded xylem would need a larger xylem area (lower *A*_s_/*A*_x_) to provide enough water for stomata loss under the same stomatal area, but the higher *A*_s_/*A*_x_ we found suggested that floating-leaved plants invested more in stomata to enhance carbohydrate assimilation rather than in functional xylem inside petioles for water transport (Gentine *et al.*, 2016; Klimešová *et al.*, 2017). We inferred that the seemingly unreasonable pattern of hydropotens on the lower (abaxial) surface of floating-leaved plants could provide additional water supply (Sculthorpe, 1967; Catian and Scremin-Dias, 2013). Indeed, though midday photosynthesis depression is common in terrestrial plants due to partial stomata closure induced by insufficient water supply, such a phenomenon has not yet been observed for floating-leaved species (Demmig-Adams *et al.*, 1989; Ritchie, 2012). Another reason for a larger *A*_s_/*A*_x_ could be related to pressurized ventilation in floating-leaved aquatic plants. At least for the two well-studied species *Nuphar luteum* and *Nymphoides peltata*, there is a well-known gas through-flow pumping system from stomata to sediment, as a particular adaptation to oxygen deficiency in sediment (Dacey, 1981; Dacey and Klug, 1982; Große, 1996), and stomatal traits were related not only to water transport (liquid or vapour water) but also for gas exchange (CO_2_ or O_2_). However, as stated above, there is extensive aerenchymatic tissue (cavities, lacunae, air spaces) in petioles of floating-leaved plants which can provide mechanical support and transport oxygen to the roots of most aquatic plants (Doležal *et al.*, 2021). Overall, the unique leaf and petiole structures of floating-leaved plants lead to different allometric relationships, which indicated a special water–carbon coupling strategy to adapt to aquatic habitats.

Meanwhile, we found similar allometric relationships between leaf area and petiole transverse area shown in Corner’s rules (Corner, 1949; Brouat *et al.*, 1998; Fajardo *et al.*, 2020), as well as between total stomatal area and petiole vascular area for floating leaves, and that between leaf and stem traits for terrestrial leaves found in Zhong *et al.* (2020). Moreover, all three slopes of these regression lines were significantly larger than 1 (hyperallometry), suggesting that the construction of leaves (or stomata) was more important than the construction of petioles (or xylem) independent of plant types. Furthermore, the construction of petioles/stems requires carbon investment that is originally captured by leaves, and therefore, we suggest that the selection pressure on leaves could be stronger than on petioles/stems. However, further analysis to compare their intercepts was not necessary because the three pairwise traits were totally different.

The allometric scaling in leaf area correlated strongly with petiole/stem transverse area, in agreement with previous studies (Gleason *et al.*, 2018; Zhong *et al.*, 2019; Filartiga *et al.*, 2022). Although Wen *et al.* (2020) stated that allometry should be considered as an adaptation of hydraulic processes in terrestrial plants, we argue that the clear pattern described by allometric equations for these aquatic plants might not only be constrained by hydraulic process, but also related to other biological processes. For example, compared with petiole xylem area, the petiole of floating leaves (i.e. petiole transverse area) not only provides water supply but also faces hydraulic drag from the aquatic environment, which may equate to the structural role that xylem has in terrestrial plants, and the petiole vasculature includes the phloem area and transports nutrient and carbohydrate, which may explain the similar scaling patterns with terrestrial plants. The slope of the allometric relationship between leaf area and petiole transverse area was >1, suggesting a larger allocation of leaf area per petiole transverse area. We propose that across plant species (both aquatic and terrestrial), additional positive selection occurred in the development of the stomata area compared with the construction of the petiole/stem area. However, as the petiole vascular area in aquatic plants and stem xylem area in terrestrial woody plants are not anatomically identical, we need to investigate additional lineages, such as terrestrial herbaceous plants, with similar vascular bundle structures in the stems to clarify the mechanisms of such similar scaling patterns. The origin of allometric scaling in plants and animals has been debated heatedly (Beuchat, 1997; West *et al.*, 1997; Hu *et al.*, 2021; Koyama and Smith, 2022; Santoro and Calzada, 2022), and our study can act as a case study to show various trait coordination in leaf and petiole (or stem) between aquatic and terrestrial plants.

A recent study on terrestrial shrubs and trees also discovered no phylogenetic conservation on these petiole traits (e.g. petiole transverse area, xylem diameter) (Filartiga *et al.*, 2022). The significant scaling relationships between total stomatal area and petiole xylem (or vascular) area in floating-leaved plants did not change after considering phylogeny. This can be explained by the divergent evolution of floating-leaved plants. As a typical life form in floating-leaved angiosperms (excluding one fern species, *Marsilea quadrifolia*), floating-leaved species have usually been considered to have evolved in a parallel way and widely distributed in basal angiosperms, monocots and eudicots (The Angiosperm Phylogeny Group, 2016). We found that most stomatal traits are strongly correlated with petiole vascular area, whether including phylogenetic relationships or not, which agrees with the findings of the phylogenetically independent hydraulic coordination between stomatal and stem traits (Liu *et al.*, 2015). However, the strength of the correlation between stomatal size (individual stomatal area) and petiole vascular area (xylem) switched from significance to insignificance after taking into consideration the phylogenetic signal (Table 1). A similar switch was also been observed by Liu *et al.* (2015), probably due to the phylogenetic background across species disturbing the conventional correlations by adding more variance into the models. Additionally, stomatal size was negatively correlated with stomatal density, which paralleled previous studies showing a trade-off between stomatal size and stomatal density (Franks and Beerling, 2009; Milla *et al.*, 2013). Accordingly, the strong correlation between petiole vascular area and stomatal density, rather than stomatal size, implied that the regulating mechanisms related to stomatal conductance were more closely related to growing dense or scattered stomata than forming different sized stomata (Franks and Beerling, 2009).

In summary, the allometric scaling between plant traits as a cost-effective method is common to both terrestrial (mainly for water transport) and aquatic plants (various physiological processes). However, the different slopes (lower) of the relationship between total stomatal area and petiole xylem area of floating-leaved plants than that of terrestrial plants indicate a different water–carbon coupling strategy. Moreover, the larger intercept of the regression between these two traits of floating-leaved plants than that of terrestrial plants suggests that either different (lower) water use efficiency in floating-leaved plants compared to terrestrial plants or sufficient water supply ensures that floating-leaved plants can capture more carbon. The current study provides new insight from the perspective of allometric scaling between leaf and petiole traits to understand the unique adaptation strategies of floating-leaved species compared with terrestrial woody species.

## SUPPLEMENTARY DATA

Supplementary data are available online at https://academic.oup.com/aob and consist of the following. Table S1: Details for the chosen 31 species/varieties in this study. Table S2: Standardized major axis regressions and comparisons between floating-leaved plants and terrestrial plants for leaf and petiole/stem traits. Table S3: Phylogenetic analysis of leaf and petiole traits. Figure S1: The relationship between petiole xylem and vascular areas across ten floating-leaved plants. Figure S2: Relationships among stomatal density and stomatal size across 31 floating-leaved plants, and stomatal density and petiole xylem area across ten species.

mcad007_suppl_Supplementary_Figures

mcad007_suppl_Supplementary_Tables
