# Supplementary material for: Allometric relationships between leaf and petiole traits across 31 floating-leaved plants reveal a different adaptation pattern from terrestrial plants
Source: Ann Bot. 2023 Jan 19;131(3):545–52. doi: 10.1093/aob/mcad007 (PMC10072084; doi:10.1093/aob/mcad007)
Supplement: mcad007_suppl_Supplementary_Figures [file mcad007_suppl_supplementary_figures.doc]

**Table S1.** Details information for the chosen 31 species/varieties in this study. Y in the Petiole xylem area column stands for the species/variety measured with petiole (and vascular) xylem area, N for the vascular area only. F in the Heterophyll column refers to the mature plants that usually have floating leaves, F-E for both floating and emergent (aerial) leaves, S-F for with both submerged and floating leaves, and S-F-E for all three-type leaves. The phylogenetic group is classified into Fern, Monocots, Eudicots and ANA (basal angiosperms). Due to the shortage of experimental plants, only four leaves of *Euryale ferox* Salisb., three leaves of *Victoria cruziana* A.D. Orb., and two of *Caldesia grandis* Sam. were determined.

| **ID** | **Family** | **Taxon** | **Replicate number** | **Petiole xylem area** | **Heterophyll** | **Phylogenetic group** |
| --- | --- | --- | --- | --- | --- | --- |
| 1 | Cabombaceae | *Brasenia schreberi* J. F. Gmel. | 10 | N | F | ANA |
| 2 | [Cabombaceae](http://www.theplantlist.org/1.1/browse/A/Cabombaceae/) | *Cabomba caroliniana* A. Gray | 10 | N | S-F | ANA |
| 3 | [Nymphaeaceae](http://www.theplantlist.org/1.1/browse/A/Nymphaeaceae/) | *Euryale ferox* Salisb. | 4 | N | F | ANA |
| 4 | Nymphaeaceae | *Nuphar japonica* DC. | 11 | N | F | ANA |
| 5 | Nymphaeaceae | [*Nuphar pumila* (Timm) DC.](http://www.theplantlist.org/tpl1.1/record/kew-2383377) | 5 | Y | S-F-E | ANA |
| 6 | [Nymphaeaceae](http://www.theplantlist.org/1.1/browse/A/Nymphaeaceae/) | *Nymphaea alba* L. | 15 | Y | F | ANA |
| 7 | [Nymphaeaceae](http://www.theplantlist.org/1.1/browse/A/Nymphaeaceae/) | [*Nymphaea candida* C. Presl](http://www.theplantlist.org/tpl1.1/record/kew-2384823) | 10 | N | F | ANA |
| 8 | [Nymphaeaceae](http://www.theplantlist.org/1.1/browse/A/Nymphaeaceae/) | *Nymphaea* ‘Huangying’ | 5 | Y | F | ANA |
| 9 | [Nymphaeaceae](http://www.theplantlist.org/1.1/browse/A/Nymphaeaceae/) | *Nymphaea mexicana* Zucc. | 5 | Y | F | ANA |
| 10 | [Nymphaeaceae](http://www.theplantlist.org/1.1/browse/A/Nymphaeaceae/) | *Nymphaea tetragona* Georgi | 11 | N | F | ANA |
| 11 | [Nymphaeaceae](http://www.theplantlist.org/1.1/browse/A/Nymphaeaceae/) | *Nymphaea* 'Wanvisa' (H) | 5 | Y | F | ANA |
| 12 | [Nymphaeaceae](http://www.theplantlist.org/1.1/browse/A/Nymphaeaceae/) | *Nymphaea* ‘Zhuguang’ | 5 | Y | F | ANA |
| 13 | [Nymphaeaceae](http://www.theplantlist.org/1.1/browse/A/Nymphaeaceae/) | *Victoria cruziana* A.D. Orb. | 3 | N | F | ANA |
| 14 | [Alismataceae](http://www.theplantlist.org/1.1/browse/A/Alismataceae/) | *Caldesia grandis* Sam. | 2 | N | F | Monocots |
| 15 | [Alismataceae](http://www.theplantlist.org/1.1/browse/A/Alismataceae/) | *Caldesia parnassifolia* (L.) Parl. | 10 | N | F | Monocots |
| 16 | [Alismataceae](http://www.theplantlist.org/1.1/browse/A/Alismataceae/) | *Hydrocleys nymphoides* (Humb. & Bonpl. ex Willd.) Buchenau | 10 | N | F-E | Monocots |
| 17 | Alismataceae | *Sagittaria natans* Pall. | 10 | N | S-F | Monocots |
| 18 | Aponogetonaceae | *Aponogeton lakhonensis* A. Camus | 11 | N | F | Monocots |
| 19 | [Hydrocharitaceae](http://www.theplantlist.org/1.1/browse/A/Hydrocharitaceae/) | *Hydrocharis dubia* (Blume) Backer | 10 | Y | F-E | Monocots |
| 20 | [Hydrocharitaceae](http://www.theplantlist.org/1.1/browse/A/Hydrocharitaceae/) | *Ottelia cordata* (Wall.) Dandy | 13 | N | S-F | Monocots |
| 21 | [Hydrocharitaceae](http://www.theplantlist.org/1.1/browse/A/Hydrocharitaceae/) | *Ottelia ovalifolia* (R. Br.) Rich. | 10 | Y | F | Monocots |
| 22 | [Potamogetonaceae](http://www.theplantlist.org/1.1/browse/A/Potamogetonaceae/) | *Potamogeton distinctus* A. Benn. | 10 | N | F | Monocots |
| 23 | [Potamogetonaceae](http://www.theplantlist.org/1.1/browse/A/Potamogetonaceae/) | *Potamogeton natans* L. | 11 | N | F | Monocots |
| 24 | [Lythraceae](http://www.theplantlist.org/1.1/browse/A/Lythraceae/) | *Trapa incisa* Siebold & Zucc*.* | 10 | N | F | Eudicots |
| 25 | Menyanthaceae | *Nymphoides hydrophylla* (Lour.) Kuntze | 11 | N | F | Eudicots |
| 26 | Menyanthaceae | *Nymphoides indica* (L.) Kuntze | 10 | N | F | Eudicots |
| 27 | Menyanthaceae | *Nymphoides peltata* (S. G. Gmel.) Kuntze | 10 | Y | F | Eudicots |
| 28 | [Nelumbonaceae](http://www.theplantlist.org/1.1/browse/A/Nelumbonaceae/) | *Nelumbo nucifera* Gaertn. | 10 | Y | F-E | Eudicots |
| 29 | [Pedaliaceae](http://www.theplantlist.org/1.1/browse/A/Pedaliaceae/) | *Trapella sinensis* Oliv. | 11 | N | F | Eudicots |
| 30 | [Polygonaceae](http://en.wikipedia.org/wiki/Polygonaceae) | *Polygonum amphibium* L. | 10 | N | F-E | Eudicots |
| 31 | [Marsileaceae](http://www.theplantlist.org/1.1/browse/P/Marsileaceae/) | *Marsilea quadrifolia* L. | 10 | N | F-E | Fern |

**Table S2.** Phylogenetic analysis on leaf and petiole traits.

| Plant traits | *K* | PIC.variance.obs | PIC.variance.rnd.mean | PIC.variance.P | PIC.variance.Z |
| --- | --- | --- | --- | --- | --- |
| Stomatal size | 0.411 | 3538.62 | 10957.29 | 0.028 | -0.845 |
| Stomatal density | 0.256 | 845.20 | 1536.28 | 0.329 | -0.424 |
| Stomatal area unit | 0.420 | 0.00 | 0.00 | 0.057 | -1.023 |
| Total stomatal area | 0.219 | 4079456.29 | 6796992.03 | 0.551 | -0.273 |
| Leaf area | 0.212 | 27737.53 | 52245.18 | 0.531 | -0.329 |
| Petiole transverse area | 0.289 | 8.05 | 18.04 | 0.326 | -0.453 |
| Petiole vascular area | 0.212 | 0.06 | 0.09 | 0.527 | -0.296 |
| Petiole xylem area | 0.876 | 0.00 | 0.02 | 0.087 | -0.572 |

**Table S3.** Standardized major axis (SMA) regressions and comparisons between floating-leaved plants and terrestrial plants for leaf and petiole/stem traits. SMA was conducted with the ‘SMATR’ package to quantify allometric parameters (slopes, intercepts and correlation coefficients) of pairwise traits across floating-leaved plants and terrestrial plants (Warton et al., 2006). The ‘SMATR’ package used for SMA regressions was also used for testing slope different from 1, slopes between types (floating-leaved plants and terrestrial plants). All traits were log-transformed before analysis. Significance levels: *p* < 0.05 = *; *p* < 0.01 = **; *p* < 0.001 = ***.

| y | x | r^2^ | *p* | *slope* | intercept | H0: slope not different from 1 (*p*) | y ~ x + type | |
| --- | --- | --- | --- | --- | --- | --- | --- | --- |
|  |  |  |  |  |  |  | H0: slopes are equal (*p*) | H0: no difference in intercept (*p*) |
| Leaf area | Petiole transverse area | 0.83 | *** | 1.24 | 0.82 | *** | 0.12 | / |
| Total stomatal area | Petiole vascular area | 0.78 | *** | 1.42 | 3.17 | *** | 0.65 | / |
| Total stomatal area | Petiole xylem area | 0.68 | *** | 0.77 | 3.4 | ** | *** | *** |
