# Supplementary material for: Allometric relationships between leaf and petiole traits across 31 floating-leaved plants reveal a different adaptation pattern from terrestrial plants
Source: Ann Bot. 2023 Jan 19;131(3):545–52. doi: 10.1093/aob/mcad007 (PMC10072084; doi:10.1093/aob/mcad007)
Supplement: mcad007_suppl_Supplementary_Tables [file mcad007_suppl_supplementary_tables.doc]

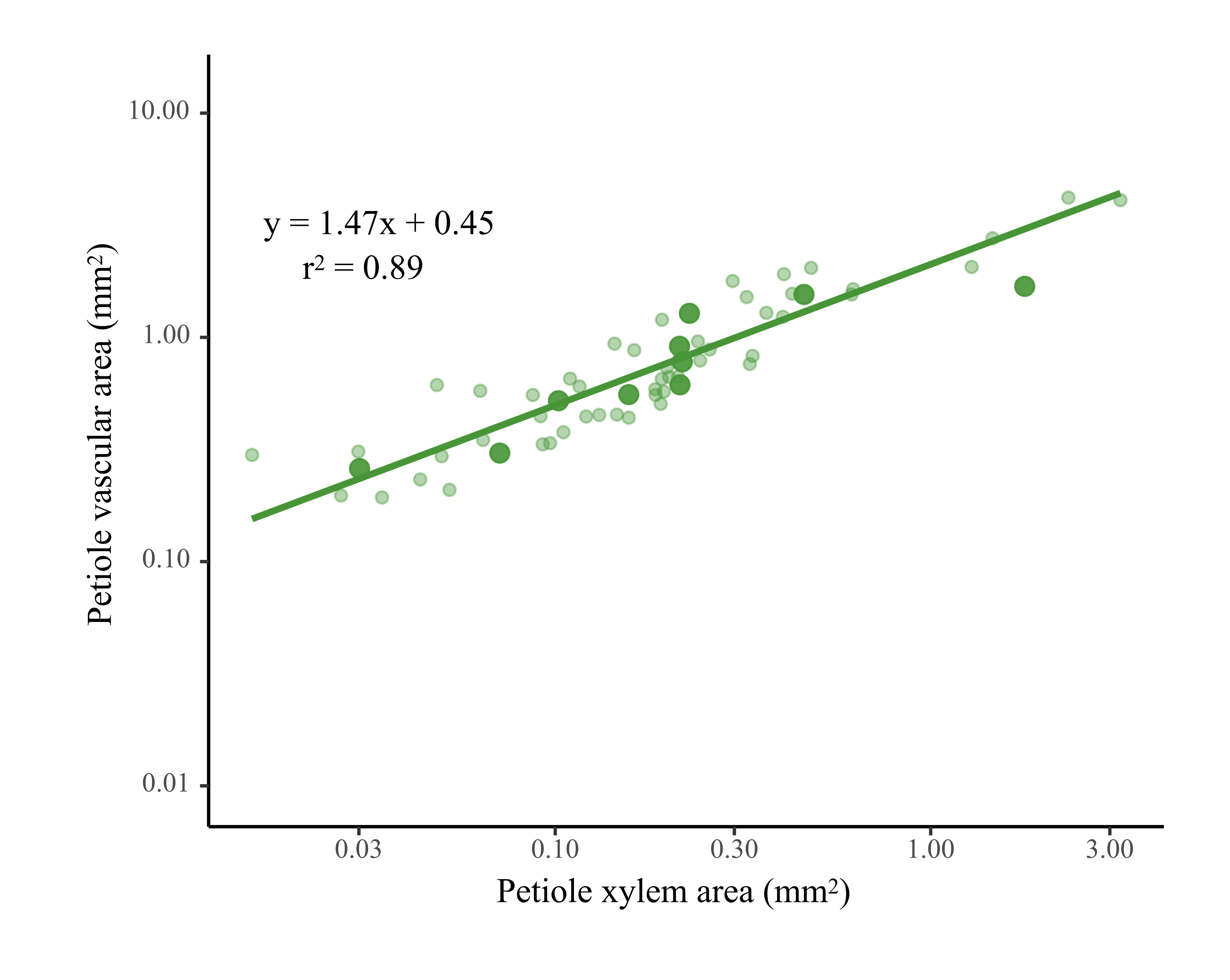


**FIG. S1.** The relationship between petiole xylem and vascular areas across ten floating-leaved plants. The big green dots represent the mean value for each species in floating-leaved plants. Both X-axis and Y-axis were log-transformed.


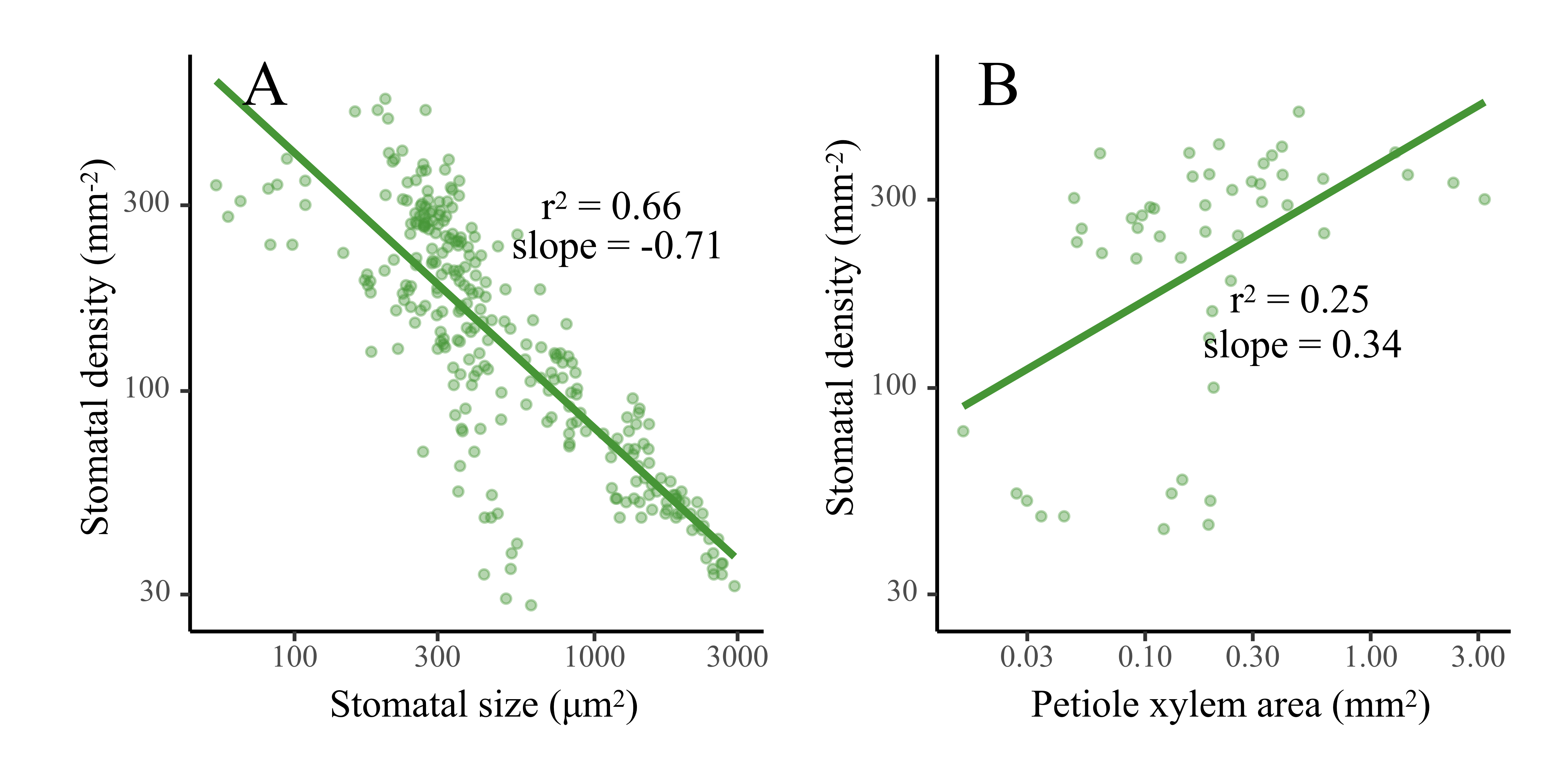


**FIG. S2.** Relationships among stomatal density and stomatal size across 31 floating-leaved plants (A), stomatal density and petiole xylem area across ten species (B). Both X-axis and Y-axis were log-transformed.
